# Role of stromal-derived factor-1<alpha>/CXCR4 in neo-intimal repair

**DOI:** 10.5830/CVJA-2010-075

**Published:** 2011-11

**Authors:** J Sheng, W-W Cai, S-Q Wang, N-Y Fang, J-J Wu

**Affiliations:** Department of Geriatrics, 9th Hospital, Shanghai Jiao-Tong University School of Medicine, Shanghai, China; Department of Geriatrics, 9th Hospital, Shanghai Jiao-Tong University School of Medicine, Shanghai, China; Department of Geriatrics, 9th Hospital, Shanghai Jiao-Tong University School of Medicine, Shanghai, China; Department of Geriatrics, Ren-Ji Hospital, Shanghai Jiao-Tong University School of Medicine, Shanghai, China; Shanghai Tissue Engineering Laboratory, Shanghai Jiao-Tong University School of Medicine, Shanghai, China

**Keywords:** percutaneous transluminal coronary angioplasty, percutaneous coronary intervention, restenosis, chemokine, stromal cell-derived factor-1<alpha>, CXCR4

## Abstract

**Abstract:**

Neo-intimal hyperplasia is one of the major causes of restenosis in which stromal cell-derived factor-1<alpha> (SDF-1α) and its receptor CXCR4 play an important role. In a rat common carotid artery balloon injury model, the number of CD34^+^CXCR4^+^ cells was significantly increased immediately after injury (*p* < 0.01), followed by a gradual decrease to baseline seven days after the injury. Furthermore, the plasma (SDF-1α) level was markedly elevated, and peaked 24 hours after injury (*p* < 0.01), followed by a rapid decrease to baseline level seven days after the injury. In the injured common carotid artery, the mRNA expression of (SDF-1α) was elevated immediately after injury, followed by a gradual decline, but that of CXCR4 was increased four days after injury.

Immuno-histochemistry displayed CXCR4-positive staining one day after injury, which then gradually increased and continued for at least one month. In addition, administration of AMD3100 (200 ng/kg, i.p.), a CXCR4 antagonist, did not affect the number of CD34^+^CXCR4^+^ cells, the elevated level of plasma (SDF-1α) and expression of (SDF-1α) mRNA. The expression of CXCR4 mRNA and protein however was markedly decreased, and detectable CXCR4-positive cells occurred four days after injury, followed by a decreased intensity of staining. We also found that, three months after balloon injury, stenosis of the carotid artery intima in the group that received AMD3100 was significantly less than in the untreated group (*p* < 0.05). Therefore, (SDF-1α)/CXCR4 played a crucial role in the intimal hyperplasia, and restenosis may have be attenuated after inhibition of CD34^+^CXCR4^+^ cells in the intima.

## Abstract

Percutaneous transluminal coronary angioplasty (PTCA) and percutaneous coronary intervention (PCI) are effective techniques in the treatment of cardiovascular diseases, including acute myocardial infarction (AMI). But a relatively high incidence of restenosis after PTCA or PCI is a major problem, causing failure of treatment. Neo-intimal hyperplasia after vascular injury plays an important role in post-treatment restenosis, which is mainly caused by excessive cell deposition in the neo-intima. Therefore, blocking cell deposition may be a promising strategy in the prevention of post-injury restenosis.

Stromal cell-derived factor-1 (SDF-1) is one of the chemokines that belong to the intercrine family and is officially designated as chemokine (C-X-C motif) ligand 12 (CXCL12).^1^ The receptor for this chemokine is CXCR4, which was previously called fusin. SDF-1 is a strong chemo-attractant in directing progenitor cell trafficking, cell migration and angiogenesis.^2^,^3^ It has been confirmed that the expression of SDF-1 was elevated in the peri-infarct area after AMI,^4^ which recruited CXCR4^+^ cells to participate in myocardial repair and angiogenesis.

In humans and rats, both SDF-1 and CXCR4 show a high degree of sequence homology. In rats, the SDF-1 gene encodes three splice variants: α, β and γ. In the present study, a rat carotid artery balloon injury model was used to mimic human coronary neo-intimal repair after PTCA or PCI. By investigating the expression of SDF-1α and CXCR4 after injury and using a CXCR4 antagonist (AMD3100) to block the interaction between SDF-1 and CXCR4, we found that SDF-1α/CXCR4 played a critical role in neo-intimal hyperplasia.

## Methods

A total of 156 male Sprague Dawley rats weighing 286 ± 14.3 g were purchased from the SLAC Laboratory Animal Co, Ltd, Shanghai, China, and housed in a temperature-controlled environment (22–24°C) with a 12-h light–dark cycle. The local ethics committee of the School of Medicine, Shanghai Jiao-Tong University (No. 0708253) approved all procedures.

The rats were randomly divided into three groups: a control group (group C; *n* = 12), a surgical group (group S; *n* = 72), and the AMD3100 treatment group (group A, *n* = 72). The rats in groups S and A were further divided into six sub-groups (*n* = 12 per group).

The rats were sacrificed as follows. Groups S_0_ and A_0_ were sacrificed 30 min after surgery, groups S_1d_ and A_1d_ one day after surgery, groups S_4d_ and A_4d_ four days post surgery, groups S_7d_ and A_7d_ seven days after surgery, groups S_1m_ and A_1m_ one month after surgery, and groups S_3m_ and A_3m_ three months post surgery.

The rat common carotid artery balloon injury model was carried out in groups S and A as previously described,^5^ and rats in the control group underwent a sham operation. Briefly, the rats were intraperitoneally anaesthetised with 2.5% pentobarbital sodium (40 mg/kg) and fixed in the supine position. A midline incision was made in the neck, and then the left common carotid artery and the bifurcation of the internal and external carotid arteries were exposed. A V-shaped incision was made on the external carotid artery followed by insertion of a 2F thrombotic balloon catheter (Edward Life Sciences, USA) deeply into the common carotid artery. The balloon was dilated by infusing ~ 0.10–0.15 ml of normal saline. The catheter was subsequently drawn back to cause damage to the intima. Then normal saline was withdrawn and the catheter was again pushed into the common carotid artery. The procedure was performed twice in order to completely remove the intima. Finally, the incision was sutured and the rats were given free access to food and water.

The rats in group A were intraperitoneally injected with 200 ng/kg/d AMD3100 (octahydrochloride, Sigma, USA) immediately before surgery for five consecutive days. The rats in group C were sacrificed two weeks later and those in the other groups were killed at the designated time. The left common carotid arteries were removed and rinsed with normal saline. Part of the artery was stored at –80°C for detection of mRNA or protein expression (*n* = 6), and the remainder was fixed for immunohistochemistry (*n* = 6).

## Flow cytometric analysis

The peripheral blood (300 μl) was incubated with FITC-conjugated anti-mouse CD34 (eBioscience, USA) and phycoerythrin-conjugated anti-human CXCR4 (eBioscience, USA) monoclonal antibodies for 30 min at 4°C (*n* = 12 per group). The cells were double-labelled with CD34 and CXCR4. The red blood cells and platelets were subsequently lysed in erythrocyte lysis buffer for 15 min, followed by centrifugation and washing.

The cells were then re-suspended in phosphate-buffered saline (PBS) and analysed on an FACS Caliber flow cytometer (BD FACSCalibur, America).^6^ Isotype-matched FITC-conjugated and phycoerythrin-conjugated antibodies (eBioscience, USA) were used as controls. The number of CD34^+^CXCR4^+^ cells was presented as the absolute number in a total of 50 000 leukocytes.

## Enzyme-linked immunosorbent assay of plasma SDF-1α

The plasma level of SDF-1a was determined by the enzyme-linked immunosorbent assay (ELISA) using an ELISA kit (R&D system, USA) according to manufacturer’s instructions.

## Real-time polymerase chain reaction analysis of SDF-1α and CXCR4

Total RNA was extracted from the injured arteries. For synthesis of cDNA, 1 μg of total RNA was reverse-transcribed with Promega RT system. Then the transcribed cDNA was amplified by polymerase chain reaction (PCR) (T3000 PCR instrument, Biometra, Germany) with specific primers as follows:

SDF-1α forward: 5′- CCAATCAGAAATGGGAACAAGA-3′, reverse: 5′- GTAGGAGGCTTACAGCACGAA-3′ (381 bp); CXCR4 forward: 5′- GTGGGCAATGGGTTGGTAAT-3′, reverse: 5′- GGTGGCGTGGACAATGGCAAGGTAG-3′ (267 bp).

The primers were synthesised by Shanghai Sangon Biological Engineering Technology & Services Co, Ltd (Shanghai, China). Reactions involved 10 min at 95°C, 40 cycles at 95°C for 15 sec, and then 60°C for one min. The products of PCR were detected with 1.8% agarose electrophoresis and visualised under a gel imaging and analysis system (Alpha FluorchemTM8900, USA).

## Western blot analysis

The artery tissues were lysed in radio-immunoprecipitation assay (RIPA) buffer (*n* = 6 per group). The protein concentration in the supernatant was measured spectrophotometrically at 595 nm. Forty mg of protein was loaded onto SDS polyacrylamide gel for electrophoresis (Invitrogen, China) and transferred to PVDF membranes (Millipore, USA).

The membrane was incubated with anti-CXCR4 antibody (1:500; rabbit anti-mouse; eBioscience, USA) and anti-<beta> actin antibody (1:1000; goat anti-mouse, Santa Cruz, USA) overnight at 4°C. The membrane was then incubated with secondary antibodies (donkey anti-rabbit antibody, 800DX 1:5000, eBioscience, USA; donkey anti-goat antibody, 700DX 1:2000 Sigma, USA) for one hour, followed by detection with an infrared fluorescence imaging and analysing system (Odyssey v1.2) (FIAS, Odyssay LI-COR USA).

## Immuno-histochemistry

Sections were then treated with 1.5% peroxide to inactivate peroxidase activity, followed by blocking with 3% bovine serum albumin. These sections were subsequently incubated with anti-CXCR4 antibody (1:250, eBioscience, USA) overnight at 4°C. The sections were then incubated with biotin-conjugated secondary antibody (1:500; Sigma, USA) or immunoglobulin G (1:500; Santa Cruz, USA).

The sections were stained with haematoxylin/eosin (H&E), dehydrated and mounted. H&E staining was performed on other sections from each group (*n* = 6) to observe the intimal change after balloon injury. The thickness of the intima was determined using the Image 45 pro analysis program.

## Statistical analysis

Experiments were performed at least three times and data were presented as the mean ± standard deviation (SD). Statistical analysis was performed with SPSS version 11.0 (SPSS Inc, Chicago, IL, USA). The unpaired *t*-test was used for comparisons between two groups and one-way ANOVA between multiple groups. A value of *p* < 0.05 was considered statistically significant.

## Results

As shown in Table 1, compared with group C, the number of peripheral CD34^+^CXCR4^+^ cells in groups S and A was significantly increased immediately after intimal injury (S_0_/A_0_) (*p* < 0.01), followed by a gradual decline to baseline seven days after injury. In group A, the number of CD34^+^CXCR4^+^ cells was increased within 24 hours after intraperitoneal administration of AMD3100, followed by a rapid decline (*p* < 0.05), which may have been related to stimulation of the bone marrow by AMD3100.

**Table 1.** Peripheral CXCR4^+^CD34^+^ Cells After Intimal Injury (*x* ± SD, *n* = 12 Per Group)

	*Group C*	*Group S*						*Group A*					
		*S_0_*	*S_1d_*	*S_4d_*	*S_7d_*	*S_1m_*	*S_3m_*	*A_0_*	*A_1d_*	*A_4d_*	*A_7d_*	*A_1m_*	*A_3m_*
CXCR4^+^CD34^+^ cells	0.021 ± 0.002	1.260 ± 0.003**	0.729 ± 0.019**	0.187 ± 0.004**	0.019 ± 0.004	0.022 ± 0.001	0.020 ± 0.038	1.411 ± 0.021**^#^	0.889 ± 0.012**^#^	0.185 ± 0.005**	0.023 ± 0.004	0.022 ± 0.011	0.019 ± 0.055

**p < 0.01 vs group C; ^#^*p* < 0.05 vs group S; d = day; m = month.

As shown in Table 2, the plasma level of SDF-1α after intimal injury was markedly increased and reached a maximum one day after injury (*p* < 0.01), followed by a rapid decrease to baseline on day seven. The administration of AMD3100 did not affect the plasma level of SDF-1α.

**Table 2.** Plasma Level Of SDF-1α After Intimal Injury (*x* ± SD, ng/ml, *n* = 12 Per Group)

	*Group C*	*Group S*						*Group A*					
		*S_0_*	*S_1d_*	*S_4d_*	*S_7d_*	*S_1m_*	*S_3m_*	*A_0_*	*A_1d_*	*A_4d_*	*A_7d_*	*A_1m_*	*A_3m_*
SDF-1<alpha> (ng/ml)	0.312 ± 0.006	0.885 ± 0.022*	1.328 ± 0.009*	1.119 ± 0.013*	0.323 ± 0.005	0.320 ± 0.006	0.309 ± 0.056	0.855 ± 0.024*	1.191 ± 0.039*	1.083 ± 0.004*	0.324 ± 0.056	0.319 ± 0.012	0.303 ± 0.027

*p < 0.01 vs group C.

**Fig. 1.**
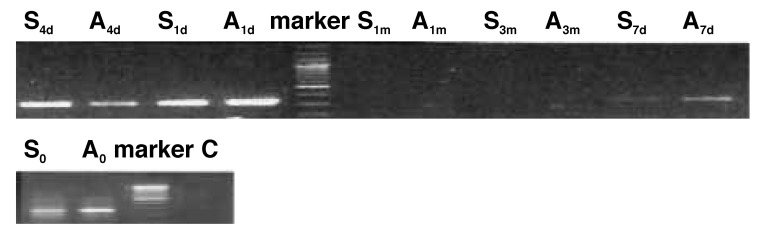
Expression of SDF-1α mRNA (381 bp) in an injured common carotid artery.

Total RNA was extracted from the injured common carotid arteries for detection of SDF-1α and CXCR4 mRNA with RT-PCR. Results showed the expression of SDF-1α mRNA in groups S and A was detectable immediately after injury, followed by a gradual decrease, but it was undetectable one month later. Administration of a CXCR4 antagonist seemed to have no effect on expression of SDF-1α mRNA (Fig. 1).

**Fig. 2.**
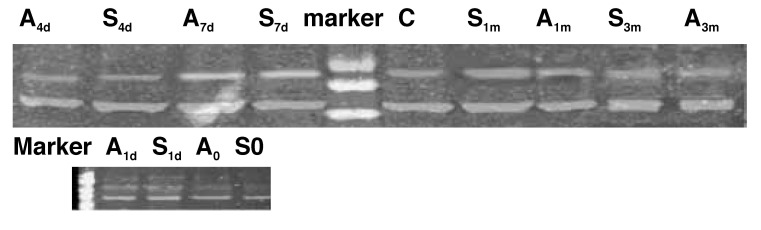
Expression of CXCR4 mRNA (267 bp) in an injured common carotid artery.

However, the expression of CXCR4 mRNA was detected four days after injury and continued for one month (Fig. 1). AMD3100 is an antagonist of CXCR4, which inhibits the interaction between SDF-1α and CXCR4, so the expression of CXCR4 mRNA was decreased in group A compared with that in group S (Fig. 2).

**Fig. 3.**
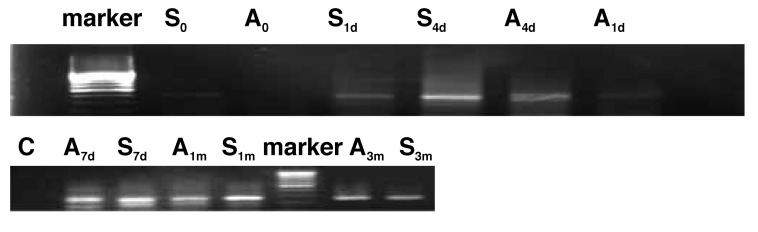
Expression of CXCR4 protein (67 kd) in an injured common carotid artery.

Western blot assay was performed to evaluate the expression of CXCR4 protein in the arteries. As shown in Fig. 3., the expression of CXCR4 protein was reduced immediately after injury, followed by a gradual increase and it lasted for three months. Results also indicated the expression of CXCR4 protein in group S was markedly higher than in group A.

**Fig. 4.**
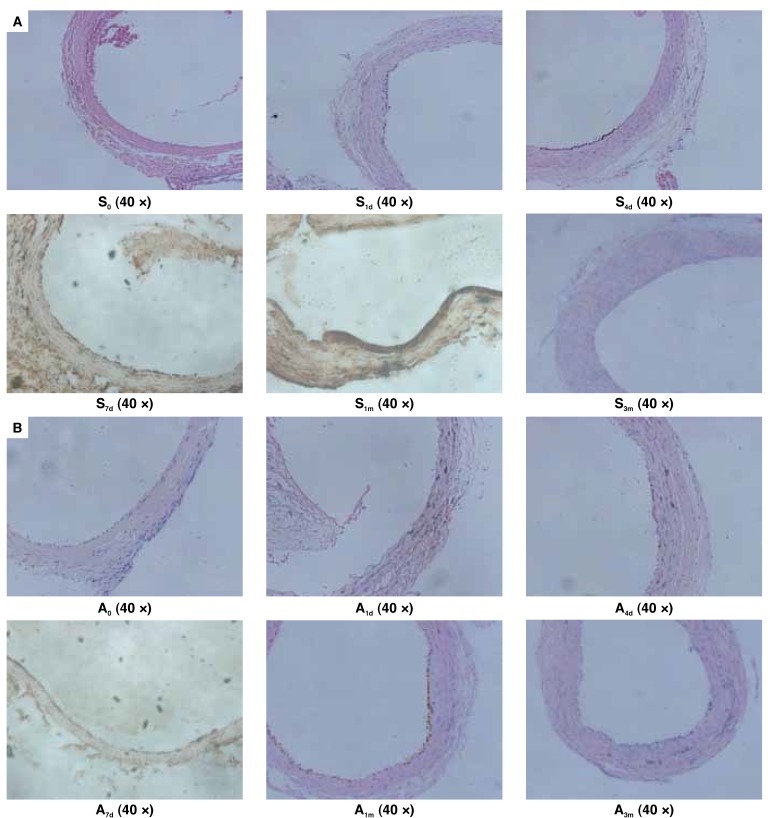
A: In group S, the positive staining for CXCR4 was detectable one day after injury and lasted for at least one month. Discontinuous brown spots were shown at the injured area. With time, the spots gradually fused to form a brown line with a slowly enhancing intensity. B: In group A, the positive staining was observed four days after injury and was sustained for one month, with a weak intensity when compared with that in group S.

Spatial localisation of CXCR4 in the injured common carotid artery was determined by immuno-histochemical assay (Fig. 4). CXCR4-positive staining was observed in the neo-intima of the common carotid artery (Fig. 4A, B). The positive staining in group S was noted as early as one day after injury, followed by a gradual increase. However, in group A, it was delayed to four days after injury, with low intensity of staining, when compared with that in group S. CXCR4 expression no longer appeared after three months in either group S or A.

**Fig. 5.**
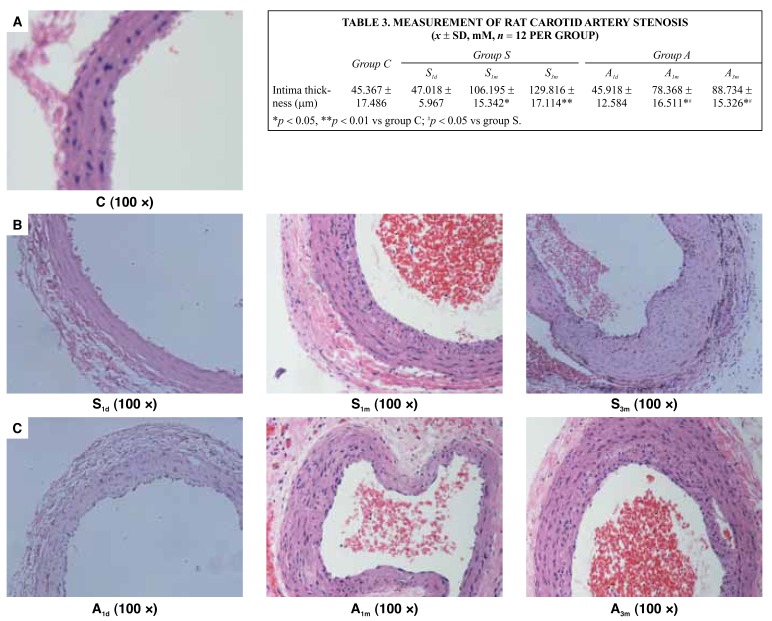
A: Normal rat carotid artery. B: The carotid artery intima of injured rats gradually underwent hyperplasia with time in group S. C: The carotid artery intima of injured rats also gradually underwent hyperplasia with time in group A, but the degree of proliferation was less than in group S.

The rat carotid artery sections one day, one month and three months after balloon injury from groups S and A were evaluated for carotid artery stenosis. After H&E staining, the sections were detected with the Image 45 pro analysis program to measure the extent of arterial intimal hyperplasia. It was found that, after one and three months, vascular intimal hyperplasia in groups S and A was significantly higher than in the control group (*p* < 0.05 and 0.01). The degree of intimal hyperplasia in group A was lower than in group S after one month (*p* < 0.05), and the difference between the two groups remained after three months (*p* < 0.05) (Table 3, Fig. 5).

**Table 3.** Measurement Of Rat Carotid Artery Stenosis (*x* ± SD, mM, *n* = 12 Per Group)

	*Group C*	*Group S*			*Group A*		
		*S^1d^*	*S^1m^*	*S^3m^*	*A^1d^*	*A^1m^*	*A^3m^*
Intima thickness (mm)	45.367 ± 17.486	47.018 ± 5.967	106.195 ± 15.342*	129.816 ± 17.114**	45.918 ± 12.584	78.368 ± 16.511*^#^	88.734 ± 15.326*^#^

*p < 0.05, **p < 0.01 vs group C; ^#^*p* < 0.05 vs group S.

## Discussion

Coronary heart disease is one of the commonest causes of death. Since the use of PTCA in the treatment of coronary atherosclerosis, numerous patients with coronary heart disease have benefited from PCI, but the relatively high incidence of restenosis after PCI has been a major problem, despite the short-term success of this technique. So far, no effective treatment strategies have been successful in preventing restenosis after PCI. Studies have demonstrated that excessive proliferation of the neo-intima plays a critical role in restenosis. Therefore, appropriate inhibition of neo-intimal proliferation may be a promising strategy in the prevention of restenosis.

Proliferation of stem/progenitor cells occurs following tissue ischaemia or damage such as acute myocardial infarction.^4^ Many cells may migrate into the ischaemic region to participate in tissue repair and angiogenesis.^7^,^8^ So far, the mechanisms underlying the progenitor cell migration to the injured site are poorly understood. Recently, stem or progenitor cell therapy has been a treatment choice for the improvement of neovascularisation and left ventricular function following acute myocardial infarction. Various types of stem cells and progenitor cells have been successfully used in the experimental acute myocardial infarction models.^9^ We speculated that, besides stem/progenitor cells being involved in intimal repair, their abnormal implantation in the neo-intima may be the main cause of vascular restenosis, in which SDF-1, an important chemokine, plays a crucial role.

SDF-1, a small (~ 8–14 kDa) pro-inflammatory chemokine,^10^ is the main regulator of cell trafficking and adhesion.^11^ It binds and activates a subset of G protein-coupled receptors with seven transmembrane domains called CXCR4, which are expressed on the surface of target cells.^12^ Although CXCR7 is also the receptor of SDF-1, CXCR4 is the only receptor known to have a definite physical role in cell trafficking, and SDF-1 is the only physical ligand for CXCR4.

The rat SDF-1 gene encodes three splice variants: α, β and γ. SDF-1α is mainly observed in the liver, spleen, kidney, heart, brain and lung and SDF-1β in the liver, kidney, spleen and thymus. SDF-1γ is predominately noted in the heart, lung, brain and peripheral nerve system.^13^ Although the levels of SDF-1γ in the heart are high, significant changes in the expression of SDF-1α were found after cardiac injury.

The interaction between SDF-1α and CXCR4 plays a crucial role in immune defense and SDF-1α is up-regulated by numerous stimuli including antigens, polyclonal stimulants, cell irritants and cytokines.^14^,^15^ In the present study, results show plasma levels of SDF-1α were significantly increased after injury, followed by a decrease to baseline four days after injury.

The number of CD34^+^CXCR4^+^ cells was markedly increased immediately after injury, followed by a gradual decline. The administration with AMD3100 increased the number of CD34^+^CXCR4^+^ cells, but no statistical significance was observed in the number of CD34^+^CXCR4^+^ cells and plasma levels of SDF-1α. Therefore, the increased plasma levels of SDF-1α and the elevated numbers of CD34^+^CXCR4^+^ cells after arterial injury may have been related to neo-intimal repair.

Numerous studies have found that SDF-1α not only stimulated haematopoietic stem cell engraftment but also recruited progenitor cells to the ischaemic region by interacting with CXCR4.^16^ After heart surgery or acute myocardial infarction, the expression of SDF-1α in the peri-injury zone was up-regulated, with profoundly increased numbers of stem/progenitor cells in the injured region.^17^,^18^

Inhibition of the SDF-1α/CXCR4 axis could partially block the recruiting of progenitor/stem cells to the injured tissues or peri-infarct myocardium.^19^ Likewise, inhibition of CXCR4 with the anti-CXCR4-antibody could also significantly reduce SDF-1α-induced adhesion of EPC to mature endothelial cells, the *in vitro* migration of EPC,^17^ and the *in vivo* recruitment of myeloid EPC to the ischaemic limb in a hind limb ischaemia model.^20^ Moreover, over-expression of SDF-1α enhanced the homing and incorporation of stem cells into ischaemic tissues.^21^,^22^ These findings support the notion that SDF-1α played a crucial role in the recruitment of circulating or intravenously infused cells.

In this study, our results showed the expression of SDF-1α mRNA was elevated immediately after injury and reached a maximum four days later, followed by a decline to baseline seven days after injury. However, the expression of CXCR4 mRNA was increased four days after injury and reached a maximum seven days after injury, followed by a gradual decrease to baseline.

Immuno-histochemistry indicated CXCR4-positive staining was found in the neo-intima (Fig. 4A, B) of the common carotid artery after injury, followed by a gradual increase in staining intensity. After the administration of AMD3100, an antagonist against the interaction between CXCR4 and SDF-1α, the expression of CXCR4 mRNA and protein was significantly decreased. The intensity of CXCR4-positive staining was also less and the time to when CXCR4-positive staining occurred was delayed. After three months, the two groups were no longer showing CXCR4-positive staining, which may have been because the interaction between SDF-1a and CXCR4 occurred only in the early injury period.

Carotid artery hyperplasia was observed with H&E staining after balloon injury. We found that compared with the control group after one and three months, hyperplasia of the neo-intima had occurred in groups S and A (*p* < 0.05 and 0.01). The degree of intimal hyperplasia in group A was lower than in group S after one month (*p* < 0.05), and the difference between the two groups remained after three months (*p* < 0.05).

We therefore postulated that the expression of SDF-1α mRNA in the left common carotid artery was increased after injury, leading to an elevated plasma level of SDF-1α, which exerted chemotactic effects on the migration of CD34^+^CXCR4^+^ cells into the injured tissues as a result of the concentration gradient of SDF-1α. The deposited cells then took part in the neo-intimal repair or even caused restenosis.

The results demonstrated that intimal repair was closely associated with the interaction between SDF-1α and its receptor CXCR4. The elevated plasma levels of SDF-1α after injury recruited peripheral CD34^+^CXCR4^+^ cells into the damaged endothelium, thus leading to formation of the neo-intima. In the injured artery, the expression of CXCR4 mRNA and protein, as well as the CXCR4-positive staining in the neo-intima was observed to be increased.

With an antagonist (AMD3100) against the interaction between SDF-1α and CXCR4, the expression of CXCR4 mRNA and protein, as well as the CXCR4-positive staining was decreased. H&E staining also showed that after the intervention of AMD3100, the rat carotid artery neo-intimal thickness was still more than the normal control group, but thinner than in the non-intervention group. Therefore, we speculated that the SDF-1α/CXCR4 axis played an important role in neo-intimal proliferation.

## Conclusion

In this study, we investigated short- and long-term changes in the SDF-1α/CXCR4 axis in the rat common carotid artery neo-intima after injury. More studies are required to explore the longterm changes in neo-intimal proliferation after administration of AMD3100, and the specific signalling pathway involved in the SDF-1α/CXCR4 axis. This may provide useful information for prevention of restenosis after PTCA or PCI.
